# Linking Corporate Social Responsibility to Workplace Deviant Behaviors: Mediating Role of Job Satisfaction

**DOI:** 10.3389/fpsyg.2021.803481

**Published:** 2021-12-30

**Authors:** Khalid Rasheed Memon, Muhammad Zada, Bilqees Ghani, Rezwan Ullah, Mohammad Tahlil Azim, Muhammad Shujaat Mubarik, Alejandro Vega-Muñoz, Dante Castillo

**Affiliations:** ^1^Graduate School of Business, Universiti Sains Malaysia, Penang, Malaysia; ^2^Business School, Henan University, Kaifeng, China; ^3^College of Business Management, Institute of Business Management, Karachi, Pakistan; ^4^School of Management and Economics, Beijing Institute of Technology, Beijing, China; ^5^Department of Business Administration, Faculty of Economics & Administration, King Abdulaziz University, Jeddah, Saudi Arabia; ^6^Public Policy Observatory, Universidad Autónoma de Chile, Santiago, Chile; ^7^Centro de Estudios e Investigación Enzo Faletto, Universidad de Santiago de Chile, Santiago, Chile

**Keywords:** corporate social responsibility, job satisfaction, turnover intentions, prohibitive voice behavior, counterproductive work behaviors, organizational identification, social exchange theory, social identity theory

## Abstract

The purpose of this article is to present a mechanism through which the deviant work behaviors of employees can be dealt-with positively through corporate good deeds in the form of fulfilling social responsibilities. Based on the spirit of social identity theory and social exchange theory, the study explores the relationships of various deviant behaviors with corporate social responsibility (CSR) through the mediation mechanism of job satisfaction. Data were collected from 385 employees of 40 large manufacturing organizations involved in CSR activities operating in Pakistan. A self-report survey was conducted using a close-ended questionnaire. Data analysis was performed using SEM through Mplus 7. The results reveal that both internal and external CSR contribute to the reduced level of turnover intention, counterproductive work behaviors, and prohibitive voice behaviors. Job satisfaction fully mediates the relationship for internal CSR while partially mediates for external CSR. The study encourages the practitioners to avail approaches that convey the feelings of care, concern, and safety, representing internal CSR practices through diverse HR interventions, organizational support, and justice. They should also keep up the socially responsible behaviors aiming toward the larger community.

## Introduction

Employee perceptions of their working environment have a significant impact on their conduct. Negative impressions relate to deviant actions such as counter-productive work behavior, prohibitive voice behavior (PVB), or turnover intention (Colbert et al., 2004). These aberrant behaviors can have significant negative impact on companies. For example, excessive turnover leads not just to direct financial expenditures but also to decreased productivity, low morale, and service interruptions, all of which result in consumer dissatisfaction (Lu et al., 2016). Additionally, PVB includes disparaging the company, criticizing work regulations and processes, all of which erode the organization’s culture and reputation (Maynes and Podsakoff, 2014; Memon and Ghani, 2020). Similarly, counterproductive work behaviors (CWB) revolve upon violating organizational norms and regulations, such as organizational fraud, production deviance, and sabotage (Griep et al., 2020).

Corporate social responsibility (CSR) is a broad notion that encompasses both the organization’s internal and external stakeholders. It is a voluntary effort on the part of the organization to benefit its stakeholders. According to research, socially responsible firms are more likely to be viewed as distinct, valuable, desired, and a source of pride for employees (Farooq et al., 2017). The social identity theory (Tajfel and Turner, 1979), with its overarching process of self-enhancement, provides a robust paradigm for analyzing the influence of CSR on employee perceptions (Turker, 2009). When workers realize how their company contributes to society’s well-being, they develop a sense of success and a desire to engage with the organization. This relationship tends to boost their self-esteem and sense of pride (Hogg and Terry, 2000; Rupp and Mallory, 2015; Farooq et al., 2017). Thus, the social identity theory provides a rational explanation for the association between perceived CSR and employees’ positive mindsets, which results in increased job satisfaction (Azim et al., 2014; Farooq et al., 2014).

Moreover, the firm’s CSR activities lead toward flourishing social exchanges, connecting a firm and its employees. CSR literature considers CSR to be an alternative to perceived organizational support, as it encompasses all types of social exchanges, i.e., generalized, and restricted exchange (Farooq et al., 2013; Memon et al., 2020). Additionally, a well-developed CSR may be considered as a type of organizational justice in and of itself, since it promotes equitable treatment of internal and external stakeholders (Mallory and Rupp, 2016). As a result, CSR activities are linked to the cognitive, emotional, and behavioral responses of stakeholders, most notably workers (Hansen et al., 2011). CSR efforts are expected to reduce workers’ turnover intentions (Chaudhary, 2017; Farooq et al., 2019), CWBs (Mashi, 2018; Agrawal and Gautam, 2020; Hamid, 2020), and PVB (Liu et al., 2010; Ng and Feldman, 2011; Memon and Ghani, 2020). However, based on the spirit of social exchange theory, we argue that this would be an indirect impact through the mediation of job satisfaction (Blau, 1964). Thus, the current study will examine the association between firm’s CSR activities and employees’ turnover intention, CWB, and PVB, through the mediating effect of job satisfaction.

Though recently some researches have been conducted to measure the effect of CSR on employee attitudes and behaviors like employee knowledge sharing behavior (Farooq et al., 2014), affective organizational commitment (Farooq et al., 2013), OCB (Shiun and Ho, 2012; Wenbin et al., 2012; Azim et al., 2014; Memon and Ghani, 2020) employee motivation (Skudiene and Auruskeviciene, 2012), employee work engagement (Rupp et al., 2013; Zulfiqar et al., 2019; Memon et al., 2020), job performance and quality of work-life (Kim et al., 2017), employees’ promotive voice behavior (Memon et al., 2021). Almost all of these are positive work behaviors where very few of the studies have been conducted for measuring CSR’s impact on negative work behaviors whereby the findings of these research leave considerable uncertainty. Specifically, we don’t know how CSR deters the negative behaviors of employees rather leads employees to behave positively toward their organizations (Zhao et al., 2019). Further, very limited studies paid attention in considering job satisfaction as dependent or mediating variable (Azim et al., 2014; Memon and Ghani, 2020) whereas more research is required to be aware of what organizational and individual variables figure out the job satisfaction and dissatisfaction of employees (Viseu et al., 2020).

This study uses three dependent variables as employees’ deviant behaviors. These are PVB, counter-productive work behavior and turnover intentions of employees. As per our knowledge, none of the previous research have been conducted, empirically measuring the relationship between CSR and PVB, based on social exchange and identification theories; rather PVB has been studied with different constructs. Even till yet, very little research has been done on PVB as a construct itself (Maynes and Podsakoff, 2014; Memon and Ghani, 2020) whereby previously PVB doesn’t contain negative connotation in its definition and was defined differently as compared to the current study (see Liang et al., 2012; Zhang et al., 2014). Please refer section “Corporate Social Responsibility, Job Satisfaction and Prohibitive Voice Behavior (A Social Exchange Perspective)” for the exact concept of PVB as per the current study. Further, very few studies are available on counter-productive work behavior whereby these are studied through social identity theory (see Shin et al., 2017; Mahmood et al., 2020). These studies do not specifically measure internal and external CSR’s impact rather measure “perceived CSR” as a whole. Such limited studies may not present the certain findings regarding the said relationships.

Only one of these variables, i.e., employee turnover intention has been studied with CSR; however either these studies are based on social identity theory (Kim et al., 2016; Wang et al., 2017; Ng et al., 2019) or social exchange theory having different variables like organizational commitment (Farooq et al., 2019) whereas the current study would propose some new relationships based on social exchange theory as well as social identity theory with different mediating variable of job satisfaction. Further, the direct effect of CSR and employee turnover intentions (Aguinis and Glavas, 2012; Gharleghi et al., 2018) have been measured by most of the previous studies; whereas some studies have found no influence of CSR on turnover intention (de Gilder et al., 2005; Jones, 2010) due to direct effect. Therefore, there is much uncertainty among the results of these studies which invokes the need to study this relationship further.

Accordingly, the present research contributes in multiple ways. Firstly, it explores the effect of firms’ CSR activities directly and indirectly (through mediation) on least explored employee behaviors i.e., PVB, and counter-productive work behavior (Farooq et al., 2013, 2014; Maynes and Podsakoff, 2014; Memon and Ghani, 2020) and turnover intentions. Secondly, it presents job satisfaction as a mediator between CSR and employee deviant behavior which is a relatively less practiced research framework for negative work behaviors (Azim et al., 2014; Memon and Ghani, 2020). Thirdly, the study incorporates the theoretical basis of both social identity and social exchange theories together in explaining the interrelationships among the stated variables (Farooq et al., 2019; Zulfiqar et al., 2019).

Finally, the current study is prominent for research in an Asian nation such as Pakistan, as earlier researches on work behavior has primarily been undertaken in the United States, the United Kingdom, or other developed countries (Antonaki and Trivellas, 2014; Maynes and Podsakoff, 2014; Kim et al., 2017). Due to the fact that emerging nations have distinct cultural, economic, and social situations, this study will undoubtedly provide something unique to the body of knowledge. For example, in most Pakistan’s work settings, the prevalence of jobs has been stated as low. Pakistani workers are notorious for being careless with their organizations and self-interested. Additionally, they are interested in some duties that are performed exclusively for them, such as internal CSR initiatives (Farooq et al., 2013, 2014; Memon et al., 2020). Indeed, recent events in the country, such as trade and market pressures resulting from global economic meltdowns, privatization of public companies, bureaucratic dishonesty and corruption, and heightened government legislation, as well as restructuring of financial sectors, have all served as a catalyst for employee withdrawal and disengagement (Shah et al., 2010; Memon and Ghani, 2020). Furthermore, this condition is developing the outlook for doubt and anxiety among workers due to an increase in the occurrence of pay deductions and increased unemployment (Memon, 2014b; Zulfiqar et al., 2019). Despite this difficult context, examining the elements that contribute to deviant work behaviors, such as PVB, is critical and valuable to the body of knowledge.

## Literature Review

### Corporate Social Responsibility-Definitions and Dimensions

The conceptualizations of the term CSR are broader and dynamic due to its implications at the micro (individual), meso (organizational), macro (country), and supra (transnational) levels (Aguilera et al., 2007; Hansen et al., 2011; Graafland and Schouten, 2012; Marquis and Qian, 2014). CSR may be defined as “actions that appear to further some social good, beyond the interests of the firm and that which is required by law” (McWilliams and Siegel, 2001). It corresponds to a form of corporate behavioral outlook toward stakeholders (i.e., external, and internal), such as consumers, employees, and the public (Farooq et al., 2013, 2014). Much research had been done during the last quarter-century on CSR and its impact on stakeholders’ relations, firm performance, external environment, corporate citizenship (Matten and Moon, 2008; Graafland and Schouten, 2012; Ooi et al., 2020).

CSR programs may include volunteer activities or policies within the firm such as incorporating greater environmental and safety standards, employee human treatment, efforts to improve employee diversity, as well as activities outside the firm such as cause-related marketing activities, community outreach programs, generous and philanthropic contributions to local communities (Hansen et al., 2011; Zheng et al., 2014). In any case, CSR efforts are usually projected to represent an illustration of a corporation as quick to respond to the requirements of the society (Ellen et al., 2006). CSR builds up the significance of an organization’s implicit claims with its stakeholders. For instance, while an employee’s wage can be predetermined in his contract, it is difficult to specify working conditions. An organization with a good reputation of care and consideration for its employees will be able to implicitly assure superior working conditions, aiding in better recruitment (Edmans, 2012).

This study would focus on the micro level i.e., internal stakeholders (individual employees) and CSR (internal and external) activities. Internal CSR merely means internal code of conduct, health and safety programs and policies, working time and environmental policies, fair pay and benefits, redundancy, and unfair dismissals (Campbell, 2007; Basil and Erlandson, 2008; Matten and Moon, 2008; Manika et al., 2017), procedural justice and high-performance work systems (HPWS) through HR (Azim et al., 2014; Farooq et al., 2019). External CSR involves organization’s behavior toward external operations i.e., customers, local communities and business partners and environmental issues (Skudiene and Auruskeviciene, 2012).

### Job Satisfaction

Job satisfaction is one of the most discussed and key constructs in organizational behavior literature since firms accomplish their desired goals and objectives through satisfied workers. It can be defined as “the reaction of people who enjoy their work and do it well, revealing characteristics of fulfillment and pride based on a range of elements” (Moro et al., 2020). Satisfied workers are strengths to their companies, portraying amplified physical and psychological health. Dissatisfied workers have inferior quality of employment and physical and psychological well-being that can adversely manipulate corporate efficiency (e.g., decrease in consumer numbers and unfavorable word of mouth or PVB) (Viseu et al., 2020).

Firms want their employees to be satisfied and perform well by mentally and emotionally committed to their careers. On the other end, employers must treat employees’ social requirements with respect by meeting their social expectations. Accordingly, Moro et al. (2020) conducted research on U.S information technology professionals, 13 percent of them were found to be dissatisfied with their jobs in 2016 and the causes were largely unfulfilled aspirations. The literature further indicates that breakage of these expectations and job dissatisfaction results in a wide variety of unconstructive consequences, including decreased organizational trust, work frustration, increased organizational cynicism, existence in general, and enhanced intention to quit (Turnley et al., 2003; Mount et al., 2006; Antonaki and Trivellas, 2014; Memon and Ghani, 2020). Contrary to this, job satisfaction results in increased productivity as well as boosting-up of inherent humanitarian value (Oshagbemi, 2013). Further, job satisfaction decreases absenteeism (Hardy et al., 2003; Adekanmbi and Ukpere, 2020), reduces counter-productive work behaviors (Meier and Spector, 2013), enhances life satisfaction (Judge et al., 2000; Viseu et al., 2020), and OCB (Organ and Ryan, 1995; Djaelani et al., 2021), leading toward higher firm performance.

## Theoretical Framework and Hypothesis Development

Social identity (Tajfel and Turner, 1986) and social exchange (Blau, 1964) theories are two profound theoretical frameworks that underpin the relationship between organizational activities including CSR and attitudinal and behavioral reactions of employees (Cropanzano and Mitchell, 2005; Azim, 2016). In the following sections, the article discusses in detail the mechanisms portraying the relationships between CSR and employee work behaviors using both the theories while hypothesizing and building our study model.

### Corporate Social Responsibility and Job Satisfaction (An Organizational Identification Mechanism)

Organizational identification refers to an organization’s workers’ sense of belonging (Mael and Ashforth, 1992, p. 104). According to the social identity theory’s premise, employees prefer to identify with their social background (i.e., their organizational affiliation) and to classify themselves and others into various social groups by comparing their organization’s characteristics, beliefs, and behaviors to those of other organizations (Farooq et al., 2017; Ko et al., 2018). Employees evaluate their self-esteem in terms of their organization’s social position. As a result, people are more inclined to affiliate with an organization whose capabilities, ideals, and activities are desired and distinguishing in comparison to those of other organizations (Azim et al., 2014). CSR is believed to be a key determinant of workers’ perceptions of their organization’s dominance and distinctiveness (Azim, 2016). Given that CSR is a voluntary effort by the business to guarantee the well-being of diverse stakeholders, it is anticipated that these activities would be highly valued by stakeholders, particularly workers. As a result, socially responsible businesses are more likely to be perceived as unique, prestigious, attractive, and a source of pride for their personnel (Ashforth and Mael, 1989). Thus, the social identity theory provides a reasonable explanation for the link between perceived CSR and workers’ favorable attitudes toward greater levels of job satisfaction, which correlates to an individual’s total affective reaction to a range of work-related factors (Cranny et al., 1992; Azim et al., 2014).

**FIGURE 1:**
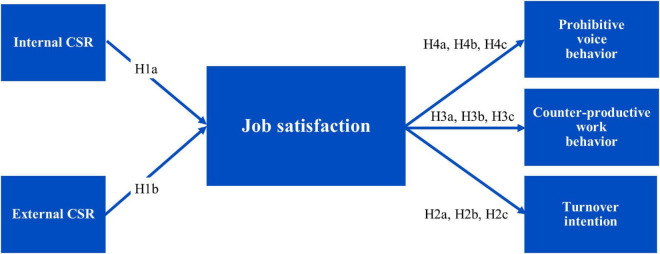
Conceptual model of the relationship between perceived CSR and employee work behaviors through mediation mechanism.

Besides, the internal component of CSR applies more directly to employee behavior (Zulfiqar et al., 2019). Cropanzano and Rupp (2003) compared CSR directed toward employees with HPWS and claimed that there was significant overlap between Human Resources Management (HRM) and the internal component of CSR. HR practices, such as pay for performance, training, and development, and empowerment have historically been closely related to corporate identification resulting in job satisfaction. These activities have certain parallels to CSR behavior aimed directly at employees. Further, previous studies demonstrate that employees may feel obliged to exhibit positive attitudes and behaviors for the organizations having good internal practices. For instance, a well thought out and sensible performance appraisal system may amplify the discernment of procedural and interactive justice among employees, resulting in heightened emotional attachment with the organization (Cropanzano and Rupp, 2003; Pham, 2020). Valentine and Fleischman (2008) found that perceived CSR has a positive implication with employee job satisfaction. Similar results are also observed by Tziner et al. (2011), Azim et al. (2014), De Roeck et al. (2014), Dhanesh (2014), and Zheng et al. (2014). Skudiene and Auruskeviciene (2012) found that internal and external CSR activities positively correlate with internal employee motivation. They observed that internal CSR was a stronger predictor of internal employee motivation than all the external CSR dimensions. Hence, we present (see Figure 1):

**Hypothesis 1a:** Employees’ perceptions of the firm’s internal CSR has direct and positive relationship with job satisfaction.
**Hypothesis 1b:** Employees’ perceptions of the firm’s external CSR has direct and positive relationship with job satisfaction.

### Corporate Social Responsibility, Job Satisfaction and Employee Turnover Intention (A Social Exchange Perspective)

The term “turnover intention” relates to an individual’s behavioral attitude toward leaving a company, whereas “actual turnover” refers to the physical process of leaving an organization (Aydogdu and Asikgil, 2011). Increased turnover not only results in financial loss, but also in decreased production, low morale, and service interruptions, creating a pool of disgruntled consumers (Lu et al., 2016). Numerous research indicates the link between turnover intention and actual departure behavior (Joo and Park, 2010). One of the most effective strategies for reducing actual turnover is to identify the elements that are likely to diminish employees’ intentions to quit the job. Employees may see organizational support, care, and concern as positive signals, preventing them from leaving the business (Srivastava and Agrawal, 2020). Internal CSR is considered an alternative to perceived organizational support in the CSR literature since it encompasses all types of social exchanges, i.e., generalized, and restricted exchanges (Farooq et al., 2013; Memon et al., 2020). Thus, it is predicted that a business’s internal CSR efforts will help reduce employee turnover intentions by linking the firm and its workers through thriving social interactions (Chaudhary, 2017; Farooq et al., 2019).

External CSR is directed at the broader community. However, it also has an influence on the feelings and emotions of employees. An organization that contributes to society’s well-being through CSR (external) generates a favorable image in the community. As a result, and consistent with the social identity approach, workers experience increased self-esteem and pride, which results in increased commitment to the business and decreased desire to leave (Hogg and Terry, 2000; Azim, 2016). De Roeck and Delobbe (2012) discovered a favorable correlation between perceived CSR (environmental CSR) and employees’ Organizational Identity.

Further, the relationship between CSR and turnover intention and other job-related attitudes and behaviors is likely to be mediated by the employees’ job satisfaction. Perceived care by the organization and enhanced self-esteem due to internal and external CSR are expected to increase the satisfaction of the employees. And the satisfied employees reciprocate through their commitment to the organization which reduces their turnover intention (Chaudhary, 2017; Mashi, 2018; Farooq et al., 2019). Previous studies support the view that there is a negative correlation between job satisfaction and employee turnover intention (Randhawa, 2007; Bright, 2008; McNall et al., 2009; Alarcon and Edwards, 2011; Aydogdu and Asikgil, 2011; Lu and Gursoy, 2013; Lu et al., 2016; Li et al., 2019). Riordan et al. (1997) found that corporate image as a proxy of social performance perceived by employees positively influences job satisfaction and negatively affects the employee turnover intention. Accordingly, we have considered the following Hypotheses.

**Hypothesis 2a:** Job satisfaction is negatively associated with employees’ turnover intention.
**Hypothesis 2b:** Internal CSR has negative relationship with employees’ turnover intention through the mediation of job satisfaction.
**Hypothesis 2c:** External CSR has negative relationship with employees’ turnover intention through the mediation of job satisfaction.

### Corporate Social Responsibility, Employees’ Job Satisfaction and Counterproductive Work Behavior (A Social Exchange Perspective)

Counterproductive work behaviors (CWB) are undesirable behaviors within the workplace. Sackett (2002) defined it as “any intentional behavior on the part of an organization member viewed by the organization as contrary to its legitimate interests.” CWB is usually described in terms of three main characteristics: (a) CWB is volitional and inspired, and thus not accidental. (b) Actions are deviant because they contradict the rules of the organization, and (c) actions are aimed at and detrimental to the organization (Griep et al., 2020). CWB can be of two types viz., (a) CWB – Organizational, which is directed to the organization, e.g., organizational fraud, public criticism of the organization, production deviance, sabotage, withdrawal, and (b) CWB–Individual which is directed at co-workers, e.g., omission, bullying, threats.

Counterproductive work behaviors is related to the emotional state of the employees. The events that promote negative emotions (sense of deprivation) are likely to increase the CWB while the events leading to positive feelings (sense of achievement) reduce such behavior. Breach of psychological contract (Griep et al., 2020), unfair treatment (Folger and Cropanzano, 2001), hostility (Judge et al., 2006), organizational injustice (Ambrose et al., 2002) are found to contribute to counterproductive behavior. In fact, such factors generate a sense of dissatisfaction among the employees, and they retaliate with CWB (Czarnota-Bojarska, 2015). Thus, job satisfaction (or dissatisfaction) is an observed proximal antecedent of CWB (Judge et al., 2006).

Corporate social responsibility activities have an effect on the attitudes and actions of both internal and external stakeholders. Numerous researches have established a link between CSR and cognitive, emotional, and behavioral responses (Cramer, 2005; Hansen et al., 2011). Internal CSR-driven organizations care about their employees’ well-being, and as a result, their employees feel cared for and safe. As a result, reciprocity develops, and workers retaliate by working diligently rather than withholding their efforts or causing harm to their business (Cropanzano and Mitchell, 2005; Mount et al., 2006). Similarly, businesses that engage in external CSR on a voluntary basis and contribute to a social cause can establish themselves as respected corporate entities. Due to their altruistic commitment to society, such firms are seen as trustworthy and helpful by existing and potential workers (Barton and Barton, 2011; Chaudhary, 2017). These attitudes and expectations establish reciprocal duties between employee and employer (Rousseau, 1989) and deter workers from engaging in negative or counter-productive work practices and behaviors (Memon et al., 2020). Given the proximity of work satisfaction to CWB, it is possible to envision that job satisfaction mediates the link between CSR and CWB (Judge et al., 2006). As a result, we examine the following possibilities.

**Hypothesis 3a:** Job satisfaction has negative relationship with employees’ counterproductive work behavior.
**Hypothesis 3b:** Internal CSR has negative relationship with employees’ counterproductive work behaviors through the mediation of job satisfaction.
**Hypothesis 3c:** External CSR has negative relationship with employees’ counterproductive work behaviors through the mediation of job satisfaction.

### Corporate Social Responsibility, Job Satisfaction and Prohibitive Voice Behavior (A Social Exchange Perspective)

Prohibitive voice behavior (PVB) generally means being vocal against organizational decisions and practices. It may take place in two forms: (a) defensive voice and (b) destructive voice (Maynes and Podsakoff, 2014). Defensive voice behavior is defined as “the voluntary expression of opposition to changing an organization’s policies, procedures, programs, and practices, even when the proposed changes have merit or making changes is necessary.” It includes, deliberately opposing the changes in working methods or SOPs (standard operating procedures) or arguing stubbornly against any proposed changes. This dimension of PVB is similar to resistance to change. However, it is active in nature. Destructive voice behavior is defined as “the voluntary expression of hurtful, critical, or debasing opinions regarding work policies, practices, procedures.” For example, harsh criticism of organizational policies, or work methods, bad-mouthing, making sarcastic comments about the organization (Maynes and Podsakoff, 2014). PVBs are likely to hurt the organizational image and damage interpersonal relationships at work (LePine and Van Dyne, 1998).

Prior studies explored the determinants of employee voice behavior in terms of individual characteristics (e.g., Crant et al., 2011), contextual factors (e.g., Stamper and Van Dyne, 2001), social exchange (e.g., Rees et al., 2013), psychological factors (e.g., Walumbwa and Schaubroeck, 2009). Several studies (Janssen et al., 1998; Fuller et al., 2007; Nikolaou et al., 2008; Landau, 2009; Crant et al., 2011; Grant, 2013; Qin et al., 2014; Wu et al., 2015) have identified personality, impression management tendencies, emotional intelligence, and cognitive style preferences, as the likely antecedents of voice behavior. Similarly, contextual factors, such as tenure (Avery et al., 2011), employee past performance (Fuller et al., 2007), leadership (Walumbwa and Schaubroeck, 2009; Hsiung, 2012), human resource management practices (Davis-Blake et al., 2003; Si and Li, 2012), voice climate (Morrison, 2011; Engemann and Scott, 2020), ethical and organizational culture (e.g., Berry, 2004; Ruiz-Palomino and Martínez-Cañas, 2014), national culture (e.g., Farh et al., 2004), and labor union (e.g., Deery et al., 2014) were found to be predictive of employee voice behavior. Some studies (e.g., Stamper and Van Dyne, 2001; Gong et al., 2010; Farndale et al., 2011; Rees et al., 2013) emphasize the social exchange relationship within the workplace as the potential cause of the voice behavior. Due to profound social exchange relationships, the employees feel more valued, recognized, and involved that consequently motivated them to engage in voice behavior. Employee voice behavior is also observed to be affected by psychological factors such as perceived risk of voicing, trust in leadership, job satisfaction, organizational identification (e.g., Janssen et al., 1998; Detert and Burris, 2007; Tangirala and Ramanujam, 2008; Walumbwa and Schaubroeck, 2009; Gao et al., 2011).

As discussed, the organizations that practice internal CSR are required to act encouragingly, demonstrating care and concern, openness and trustworthiness, involvement in goal setting, and communicate the vision with their employees through HPWS so that employees are satisfied with their jobs (Memon et al., 2018, 2021). Similarly, due to external CSR, an organization earns a reputation, and the employees consider working in those particular organizations as pride and prestige for themselves. This sense of job satisfaction, in line with the norm of reciprocity, would enable them to raise positive voice (Arnold et al., 2000; Liu et al., 2010; Ng and Feldman, 2011) while refraining them from PVB s that may damage the organization (Memon and Ghani, 2020). Therefore we present our hypothesis as:

**Hypothesis 4a:** Job satisfaction has negative relationship with employees’ prohibitive voice behavior.
**Hypothesis 4b:** Internal CSR has negative relationship with employees’ prohibitive voice behavior through the mediation of job satisfaction.
**Hypothesis 4c:** External CSR has negative relationship with employees’ prohibitive voice behavior through the mediation of job satisfaction.

## Methodology

### Sample and Procedure

The study is based on a survey managed through a self-reported questionnaire. However, the time lag technique has been used to avoid common method bias, and accordingly, temporal, and psychological separations of our variables are used (Farooq et al., 2017) our focus is on the employees of different manufacturing units in Pakistan. Their products consisted of food items, paper manufacturing, auto parts manufacturing, and cement manufacturing. These companies have larger sales volumes since Pakistan is having a population of approximately 220 million inhabitants.

The study used convenience sampling technique for collecting the data. In total 75 companies were selected based on their CSR information available through secondary data sources, especially websites implying that they are involved in CSR activities and their employees are well aware of the related concepts and activities (Farooq et al., 2013, 2014). Forty out of 75 firms agreed to participate in our research study (Food, paper, automotive parts, and cement manufacturing companies). Further, data from the non-management staff was collected who are not directly involved in CSR policy making and implementing but are the direct observer and affected by CSR activities (Rupp et al., 2006). We divided our variables into two sections and created two distinct sheets. At time 1, the first booklet assessed CSR and job satisfaction elements in addition to demographic data, whereas the second booklet assessed PVB, counter-productive work behavior, and employee turnover intentions. This data collecting procedure necessitated the division of periods ranging from 15 to 20 days. However, it stays cross-sectional because no change is anticipated over this time period (Farooq et al., 2017). Questionnaires were sent to workers of the selected businesses with the assistance of our field survey assistants. The questionnaire was delivered with a cover letter explaining the purpose of the study and obtaining approval from the employee to participate in the research. Through this procedure, we collected the data from 385 employees (Mid-level and supervisory level managers and the operative employees) completed in every aspect after period 2 whereas there were 430 employees who filled the questionnaire at period 1. So, the drop out of 45 employees occurred. The sample size N is calculated by the priori power analysis (Cohen, 1988) based on the suitable degree of power (1-β), the pre-specified degree of significance, and the magnitude of the population effect that may be seen with probability (1-β). Before a thorough study is done, *a priori* analysis can regulate the prediction potential effectively (Hager, 2006; Faul et al., 2007). There is a minimum sample size of 107, and actual power of 85 percent when using the program G*Power 3 with input parameters: medium effect, probability of Type I error α = 0.05, probability of Type II error β = 0.05 which means (1-β) = 0.95 and number of predictors = 2. Thus, the sample size of 385 is enough to justify our results. The demographic characteristics of the sampled employees are given in Table 1. It shows that the sample comprised of 280 males and 105 female respondents. Around 70% (276) of the respondents have a bachelor’s degree and above. It means most of the respondents were academically qualified enough. Among the respondents, 243 employees had less than 5 years of service with the organizations while 142 had more than 5 years. As regards, their position in the hierarchy, 181 respondents belonged to mid-level and supervisory level management and the rest were operative employees (see Table 1).

**TABLE 1:** Demographics of the respondents.

Demographics		Frequency
Age	18–28	180
	29–40	160
	41–55	45
Gender	Male	280
	Female	105
Service tenure (years)	1	46
	2	39
	3	68
	4	90
	5	66
	6 and more	76
Qualification	Below bachelors	118
	Bachelors	182
	Masters	65
	MS/Mphil	20
Management Level	Middle management	95
	Supervisor	86
	Non-managerial staff	204

### Tools and Measurements

Several tools, whose validity and reliability have already been established, were adapted, to test the model. For instance, perceived CSR was measured through the instrument, originally developed by Turker (2009). However, the same was amended by adding one additional item from Maignan and Ferrell (2000) related to charities and donations to fulfill the contextual requirements of Pakistan. A similar scale was also adopted by Farooq et al. (2013, 2014) in their studies in Pakistan. This tool includes 16 items in total having 10 items for External CSR (community, environment, and consumers). For example, “Our organization implements special social programs to minimize its negative impact on the natural environment.” Six items were used to measure Internal CSR (Employees). For example, “Our organization policies encourage the employees to develop their skills and careers.”

#### Job Satisfaction

We used a five-item perception questionnaire, developed by Hackman and Oldham (1975) and adopted from Zhang et al. (2014), to measure job satisfaction. A sample item includes: “Overall, I am very satisfied with this job.” The questionnaire has a Cronbach alpha of 0.851 “as adopted from Zhang et al. (2014).”

#### Prohibitive Voice Behavior

Prohibitive voice behavior was measured through a 10-item instrument developed by Maynes and Podsakoff (2014) as it measures the voice behavior of employees as per our operational definition i.e., voice behavior in a defensive and destructive sense. Cronbach alpha for the defensive voice was 0.92 while it was 0.93 for destructive voice.

#### Counterproductive Work Behavior

It was measured through a two-dimensional workplace deviance scale developed by Bennett and Robinson (2000). It consisted of 12 items for measuring organizational deviant behaviors that are harmful for the organization and 7 items for individual/interpersonal deviant behavior that are harmful to an individual within the organization. For instance, “Come in late to work without permission”; “Publicly embarrassed someone at work.” Their reliability scores were 0.81 and 0.78 respectively.

#### Turnover Intentions

The three-item turnover intention scale was adopted from Khatri et al. (2001) since it was developed for the Asian context. Sample item includes, “I will probably look for a new job in the next year.” Its Cronbach alpha was 0.87.

#### Demographic and Control Variables

Research imply that age, gender, service tenure, type of employment could potentially manipulate employees’ voice behavior and work behaviors (Liang et al., 2012; Zhang et al., 2014) turnover intentions (Huang and Cheng, 2012; Farooq et al., 2019) and job satisfaction (Singhapakdi et al., 2014), therefore we controlled these demographic variables in data analysis.

We adapted the instrument and translated it into Urdu (the national language of Pakistan). The translated questionnaire was then examined by 2 management research experts. We also pre-tested the instrument through 20 Executive MBA students to identify any potential problems associated with adaptation and translation. We found some minor problems regarding translation which were corrected at once. However, no such problem was found with its structure and flow. Thus, both the experts’ reviews and pre-test revealed that the questionnaire is readable and comprehensive as well as fit for our contextual requirements. (Items available at Supplementary Appendix 1).

## Findings and Results

### Assessing the Tools and Measurement (Confirmatory Factor Analysis)

Since most of the measurement tools were adopted from Western research, it was important to test their validity in the context of a developing country (Zulfiqar et al., 2019). Confirmatory Factor Analysis (CFA) was conducted to assess the convergent and discriminatory validity of all study instruments.

We established a calculation structure for all variables and covariate them in a single model. This helped us to determine the cross-loadings if any and allowed us to quantify the discriminant validity of the tools. We used Mplus 7 to test both the measurement model and the hypothesized model. The 6-factor model provided a good fit for the results (*X*^2^ = 546.82; Df = 366; *X*^2^/Df = 1.50; CFI = 0.97; TLI = 0.96; RMSEA = 0.04; SRMR = 0.03). Contrary to this, while all factors were loaded to one factor through a single factor model, it provided a poor fit with results. In a six-factor model, the typical loading factor was greater than 0.50. Those items having lesser values were deleted (Kline, 2011; see items loadings at Supplementary Appendix 1 and Table 2).

**TABLE 2:** Model fit statistic.

Model fit indices	*X* ^2^	DF	*X*^2^/DF	CFI	NFI	TLI	RMSEA
	546.82	366	1.50	0.97	0.944	0.96	0.04

*DF, Degrees of Freedom; X^2^/DF, minimum discrepancy; CFI, Confirmatory Fit Index; NFI, Normed Fit Index; TLI, Tucker–Lewis Index; RMSEA, Root Mean Square Error of Approximation.*

We have also evaluated the convergent and discriminant validity of all six latent variables using the average extracted variance (AVE) approach suggested by Fornell and Larcker (1981). The AVE values that were higher than the suggested value of 0.50 are shown in Table 3. Thus, all the constructs have high convergent validity. In addition to the adequacy and validity of the measurement tool, we have assessed the internal reliability and consistency of our measurements. The values of Cronbach Alpha (Cronbach, 1951) and Joreskog Rho were both adequately higher than the recommended value of 0.7 (Nunnally, 1978; see Table 3).

**TABLE 3:** Reliability and convergent validity.

Latent variables	# of items	Convergent validity	Cronbach’s alpha	Joreskög’s Rhô.
External CSR	6	0.65	0.79	0.78
Internal CSR	5	0.80	0.90	0.90
Job satisfaction	4	0.72	0.86	0.86
CPWB	12	0.69	0.90	0.91
TOI	3	0.82	0.85	0.85
PVB	9	0.76	0.82	0.82

*CPWB, counterproductive work behavior; TOI, turn over intentions; PVB, prohibitive voice behavior.*

For the determination of discriminant validity, we used a factor-based procedure (Fornell and Larcker, 1981). This is the most meticulous and powerful approach to solve the problems of the difference in the chi-square method (Fornell and Larcker, 1981). By using this method, we concluded that these constructs were dissimilar from each other since the square root of the average variance derived from each construct was greater than the following correlations (see Table 4). Table 4 also presents the mean, standard deviation, and correlation of all variables studied.

**TABLE 4:** Descriptive statistics correlation matrix and test of discriminant validity.

Latent variables	Mean	*SD*	1	2	3	4	5	6
External CSR	3.71	0.68	0.75					
Internal CSR	4.19	0.67	0.37**	0.89				
Job satisfaction	3.82	0.58	0.29**	0.39**	0.84			
CPWB	4.07	0.64	0.23**	0.36**	0.38**	0.83		
Turnover	3.60	0.54	0.20*	0.28*	0.38**	0.18	0.89	
PVB	4.10	0.68	0.22*	0.26**	0.32**	0.16	0.18	0.88

CPWB, counterproductive work behavior; PVB, prohibitive voice behavior. Values on the diagonal represent the square root of convergent validity. Values in the columns are the correlations between two constructs. Values denoted with (*) are significant at 0.05 and with (**) are significant at 0.01.

### Testing of Model

Hypotheses were evaluated using Mplus version 7 for structural equation modeling. The hypothesized study model provided a rather good fit with the results (*X*^2^ = 546.82; Df = 366; *X*^2^/Df = 1.50; CFI = 0.97; TLI = 0.96; RMSEA = 0.04; SRMR = 0.03). The direct results of this model are displayed in Table 5. The significance level is given in brackets whereas the values outside the brackets are standardized regression weights. Job satisfaction, CPWB, TOI, and PVB are dependent variables. According to given table the CSR has direct relationship with job satisfaction whereas the job satisfaction has direct relationship with CPWB, TOI, and PVB. The CSR (as a whole) tended to be a good predictor of job satisfaction. The internal CSR had the highest effect of 0.40 at a degree of significance of 0.001. External CSR had an effect of 0.36 at a significance level of 0.001. As a result, our hypothesis-1a and 1b were completely supported by the evidence. Further, the results revealed the negative impact of job satisfaction on employees’ CWB, PVB, and turnover intentions as shown in Table 5 that support our Hypotheses 2a, 3a, and 4a (see Table 5).

**TABLE 5:** Testing of model.

	Job satisfaction	CPWB	TOI	PVB	Hypothesis supported
External CSR	0.36 (0.000)	–	–		1b
Internal CSR	0.40 (0.000)	–	–		1a
Job satisfaction	–	–0.34 (0.001)	–0.20 (0.001)	–0.38 (0.001)	2a, 3a, 4a

*CPWB, counterproductive work behavior; TOI, turnover intention; PVB, prohibitive voice behavior.*

### Mediation of Job Satisfaction Between Corporate Social Responsibility Dimensions and Employee Work Behaviors

We analyzed the mediation of job satisfaction between CSR dimensions and Employee work behaviors in MPlus employing a structural equation modeling technique (Lacobucci et al., 2007). MPlus has an edge greater than the other statistical tools since it presents direct and indirect effects at the same time. We also considered the direct effects of independent variables on the dependent variable job satisfaction after partialing out the effect of the mediator (C-prim Path). The studies indicate that the mediation occurs when both the independent to mediator and mediator to dependent path coefficients are significant (Lacobucci et al., 2007). ^1^AB Path represents the indirect relation of CSR dimensions on employee behaviors through the mediation of job satisfaction; Values outside brackets are a standardized indirect relation obtained from the hypothesized model; these paths represent single mediation. ^2^CB’ path represents the direct relations of CSR dimensions after partialing out the impact of the job satisfaction (mediator); here also the values outside brackets are standardized regression weights obtained from the hypothesized model. The values inside the brackets of both paths are significance levels (see Table 6).

**TABLE 6:** Mediation of Job satisfaction between CSR Dimensions and Employee work behaviors.

Independent variables	Indirect relations ^1^ AB path	Direct relation ^2^ CB’ path	Proportion of mediation AB/AB + CB’	Remarks	Hypothesis supported
External CSR → TOI	–0.06 (0.01)	–0.05 (0.15)	0.06 (6%)	Partial mediation	2c
Internal CSR → TOI	–0.057 (0.02)	0.74 (0.02)	–	Full Mediation	2b
External CSR → CPWB	–0.08 (0.01)	–0.05 (0.12)	0.08 (8%)	Partial mediation	3c
Internal CSR → CPWB	–0.048 (0.02)	0.72 (0.02)	–	Full mediation	3b
External CSR → JS	–0.09 (0.01)	–0.05 (0.14)	0.09 (9%)	Partial mediation	4c
Internal CSR → JS	–0.056 (0.02)	0.78 (0.02)	–	Full mediation	4b

*Dependent variables: CPWB, counterproductive work behavior; TOI, turnover intentions; PVB, prohibitive voice behavior.*

**FIGURE 2:**
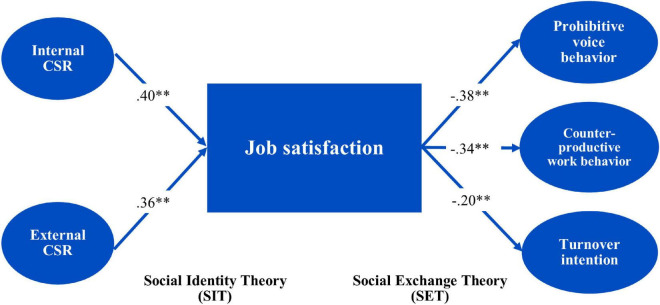
The estimated model of the relationship between perceived CSR and employee work behaviors through mediation mechanism. Values given on the paths are standardized regression coefficients. Values denoted with (^**^) are significant at 0.01.

The results shown in Table 6 indicate that our Hypotheses 2b, 3b, and 4b were fully accepted while 2c, 3c, and 4c were partially accepted since one path of these hypotheses is not significant. Indirect effects of the two dimensions of CSR were significant which shows that these two dimensions of CSR negatively affect CWB, turnover intentions, and PVB through job satisfaction. The external CSR had a direct negative effect (after partialing out the effect of mediator) on CWB, turnover intentions, and PVB which demonstrate the partial mediation in case of external CSR and full mediation in the case of internal CSR. These results portray that the indirect effect of internal CSR on employee behaviors via job satisfaction was significant at the 0.05 level whereas the indirect effect of external CSR was significant at the 0.01 level (see Figure 2).

## Discussion

This study explores the impact of CSR on deviant workplace behaviors through the mediation of job satisfaction. Specifically, it studies the impact of both internal and external CSR on turnover intention, CWB, and PVB as mediated by job satisfaction. The theoretical foundation of the study is grounded on the social identity theory and social exchange theory. Contrary to previous studies, which have focused on usual constructs for measuring the effect of CSR on, for instance, affective organizational commitment (Farooq et al., 2013; Shin et al., 2017), organizational identification and knowledge sharing behavior (Farooq et al., 2014; Wang et al., 2017; Mahmood et al., 2020), and organizational trust as mediators (Farooq et al., 2019), the current study has focused on the least focused construct of job satisfaction as mediator leading toward negative work behaviors.

The result of the study indicates that internal CSR has been the best indicator of job satisfaction leading toward a shield against employees undesirable work behaviors. The findings are consistent with the HPWS literature that stressed the use of human capital management strategies to motivate workers to be a source of competitive advantage (Farooq et al., 2019; Chen et al., 2021). Thus, the result further consolidates the existing literature, showing a positive partnership between job satisfaction and internal CSR to create a pool of motivated employees.

Our findings of, for example, Hypothesis 1 and Hypothesis 2 are compatible with other research on CSR conducted in the context of Pakistan (Farooq et al., 2013, 2014, 2019; Zulfiqar et al., 2019; Memon et al., 2020, 2021) presenting that CSR programs, contribute to the growth of trust and faith and develops the feelings of job satisfaction (Azim et al., 2014). It is found that, due to overt support for workers by presumed organizational justice (hypothesis 3 and hypothesis 4), through internal CSR and the generalized feelings of justice developed through external CSR (Farooq et al., 2017), employees in-turn, reciprocate by exhibiting their attachments to the organization in the form of a reduced level of turnover intention, PVB and CWB (Seibert et al., 2011; Singh and Singh, 2019; Memon and Ghani, 2020).

The study’s findings also indicate a clear positive impact of CSR’s external dimension on job satisfaction. This shows that CSR activities related to economic growth, grants, charities, and the commitment to social problems are also not unrelated to the employees’ feelings and emotions. This indicates that organizations’ CSR behavior is significant and vital to understanding how workers react to these organizational actions. Philanthropic contributions of the organizations add to the meaningfulness of the job and the employees feel proud of their organizations. They also view their organization as caring and responsible which eventually instigates a higher sense of emotional bondage with their organizations. Consequently, they review their relationships with the organization in a positive way.

According to the findings, compared to the external CSR, the employees are found more inclined toward internal CSR activities. This indicates that workers in Pakistan are more concerned with activities that are precisely related to them than those to others (external stakeholders). This is possibly due to the relatively poor economic status of the country. In the absence of social benefits accorded by the government, people in Pakistan largely depend on their personal income for meeting up their necessities of life. For this, job security is a prime concern for them. When the employees find that the organization is caring and concerned about them, they feel secured and become loyal to their organizations and sincere in their actions, in reciprocation. This is reflected in the result as it shows full mediation with internal CSR and partial mediation with external CSR.

## Theoretical Implications

This study proposes a model based on the huge construct of employees’ job satisfaction, developed as a result of CSR activities (external and internal) conveying the feeling of care and concern for employees, trust, and organizational justice. This empirically tested model within the context of Pakistan provides important implications for researchers.

Firstly, most of the recent studies have been done in developed countries, while our research focuses on developing country, since the data has been gathered from employees of 20 manufacturing units of Pakistan. This part of the world is predominantly a part of the developing world in terms of the global, economic, and cultural environment (Khatri et al., 2001; Memon et al., 2021). This extends the boundary conditions of the earlier studies.

Secondly, the domino effect of our research advances the underlying theories on the relationship of CSR, job satisfaction, and employees’ work behaviors in organizations. Our study finds support in the existing literature for perceived fairness at the place of work felt through firms’ CSR activities especially internal CSR, influences employees’ sense of trust and job satisfaction (e.g., Valentine and Fleischman, 2008; Seibert et al., 2011; Ugwu et al., 2014; Singh and Singh, 2019; Memon and Ghani, 2020) leading toward positive voice behavior (Liu et al., 2010; Morrison, 2011; Seibert et al., 2011; Antonaki and Trivellas, 2014; Memon et al., 2021) deterring from counter-productive work behavior (Shin et al., 2017; Mahmood et al., 2020). Similarly, we found support for CSR activities leading toward positive work behaviors deterring the negative behaviors (Farooq et al., 2013, 2014; Yu and Choi, 2014; Mallory and Rupp, 2016; Zulfiqar et al., 2019; Memon et al., 2021).

Thirdly, our study provides an interdisciplinary paradigm when it extends social psychological theories to the interpretation of an organizational phenomenon. We combine a macro-level CSR definition with micro-level employee-related variables. Rupp et al. (2006) indicate that the CSR literature lacks a comprehensive micro-level review due to over-emphasis on macro-level studies. This research thus opened a new avenue in CSR research by highlighting the role of CSR in employee attitudes and behavior (Aguinis and Glavas, 2012).

Fourthly, in comparison, the latest literature has mainly looked at the direct effect of CSR on employee attitudes. The method by which employee behavior is developed from CSR also needs to be explained. The results of the current study indicate that the CSR practices of firms become the source of employees’ job satisfaction, which in turn affects their intentions and behaviors. Explaining the sustaining mechanism, the overall implication of the study is significantly enhanced. Accordingly, we broaden and enhance recent research that discuss the influence of firms’ involvement in CSR practices on employee attitudes and behavior in the workplace.

Fifthly, previous studies on job satisfaction-CWBs (i.e., JS-CWB relationship) have fragmented results. Some of these are inclined toward negative relationships (see, for instance, Crede et al., 2007; Guo, 2012; Zhang and Deng, 2016; Malhotra and Kathuria, 2017) whereas there are instances where the said relationship i.e., JS-CWB has been reported positive or have a partial effect in decreasing CWB (see Dalal, 2005; Mount et al., 2006; Illies et al., 2009; Greenidge et al., 2014; Czarnota-Bojarska, 2015). Therefore, the said research contributes to the body of knowledge with regards to the relationship between JS-CWB and their variety of relationships whether partial or full (He et al., 2021). Moreover, the exploratory study measuring the impact of CSR activities on job satisfaction leading toward CWB is new to the body of knowledge. This would open a new horizon for researchers to study and understand the way in which CSR affects CWB through job satisfaction since previously diminutive research are available on the subject matter.

## Conclusion and Managerial Implications

Based on a developing country’s context, this paper demonstrates that both internal, as well as external CSR, have a bearing on the attitudes and behaviors of employees. It reveals that both the forms of CSR act as the distal antecedents of deviant workplace behaviors including turnover intention, counterproductive behavior, and PVB through the process of social identity and exchange relationships mediated by job satisfaction. This study further demonstrates that the internal CSR activities targeted to the employees themselves have a greater impact on employee attitudes and behaviors than those of the external CSR aimed at the society at large. These findings on the differential effects of CSR activities have multiple implications for managers and researchers.

The greater influence of internal CSR implies that Management should be concerned about responsible human resource management within the workplace (Zhuang et al., 2021). Memon et al. (2020) stress the notion that the employees are very adaptive. They analyze and perceive signals in the workplace and establish attitudes. This means that as soon as employees realize that the organization has an environment of concern and commitment to them, the employees reciprocate the caring behavior and are inclined to carry out their duties for the benefit and progress of their organization. It urges professionals and managers to convey their concern, care, and protection through several human resource interventions, organizational support through leader’s mentoring behaviors, and the communication system; since these are the most influential factors of employee motivation and instrumental in resisting employee deviant behaviors (Bedarkar and Pandita, 2014; Babalola et al., 2020; Sherf et al., 2020).

Moreover, provided that the moral development of managers is known to have an impact on CSR activities (Valentine and Fleischman, 2008), organizations should consider a wide spectrum of leadership issues in the promotion of employees (Memon, 2014a). In particular, organizations should not only focus on increasing the efficiency of workers but should also consider the degree to which managers can effectively respond to diverse work-based ethical circumstances. Companies should also develop training sessions that center on managerial ethical thought and use situation-centric vignettes and role-playing (Valentine and Fleischman, 2008).

Reciprocal relationships between external CSR and employee deviant behaviors imply that spending money for the social cause by the organizations has its spillover effects. Therefore, the managers should not consider CSR as a one-way approach of giving, it also reverts back in the form of corporate image, a satisfied pool of employees, and better organizational functioning. It is not only the demand of the larger community to perform philanthropic CSR, but also the latent expectations of the employees.

## Limitations and Directions for Future Research

This study has several shortcomings. First, since it deals with the phenomenological issue of employee feelings and emotions, data collection through a close-ended questionnaire may not reveal the true picture. Hence, in-depth interviews, focus group discussion, observation, and other qualitative approaches may be applied for better comprehension of the issue. Second, the reaction to the internal and external CSR activities of an organization by the employees is expected to be affected by demographic (age, gender, education), psychological (Personality, values) and contextual (Culture, organizational size, organizational type) factors. However, the current study does not explore the moderating effect of such variables. Therefore, future studies may pay attention to this aspect. Third, this particular study is conducted in the context of a single country (a developing country) with a relatively small number of respondents, it is, therefore, not possible to generalize the result for the developing countries, even though it offers a general understanding of the phenomenon in the context of the other Asian countries, particularly emerging South Asian countries with similar infrastructure and economic conditions. A multi-country study of a similar issue warrants better generalization. Fourthly, the study used single source data, although the common method biasness was catered through the use of time lag technique as well as marker variables and thus no such problem was found, yet future research may use multiple source data, i.e., peers/co-workers, managers may be asked to report employee deviant behaviors. Finally, one major limitation of the study is its sampling procedure. It applies convenient sampling that seriously limits the representativeness of the sample. Data collection through random sampling will overcome the weakness. However, the study satisfies all the statistical requirements for a correct interpretation of the analyzed data set.

## Data Availability Statement

The original contributions presented in the study are included in the article/Supplementary Material, further inquiries can be directed to the corresponding author/s.

## Ethics Statement

Ethical review and approval was not required for the study on human participants in accordance with the local legislation and institutional requirements. The patients/participants provided their written informed consent to participate in this study.

## Author Contributions

KM, BG, and RU contributed to conception and design of the study. MA organized the database. MM performed the statistical analysis. KM, BG, RU, MA, and MM wrote the first draft of the manuscript. MZ, AV-M, and DC wrote sections of the manuscript. All the authors contributed to manuscript revision, read, and approved the submitted version.

## Conflict of Interest

The authors declare that the research was conducted in the absence of any commercial or financial relationships that could be construed as a potential conflict of interest.

## Publisher’s Note

All claims expressed in this article are solely those of the authors and do not necessarily represent those of their affiliated organizations, or those of the publisher, the editors and the reviewers. Any product that may be evaluated in this article, or claim that may be made by its manufacturer, is not guaranteed or endorsed by the publisher.

## References

[B1] Adekanmbi F. P. Ukpere W. I. (2020). Individual substance abuse, perceived workplace fairness and organisational factors as predictors of absenteeism among civil servants in Oyo State. *Psychol. Educ.* 57 309–323.

[B2] Agrawal P. Gautam O. (2020). “The effects of leaders’ behavior on job satisfaction, organizational citizenship behavior, deviant behavior, and job performance of employees,” in *Analyzing Workplace Deviance in Modern Organizations*, ed Sharma N. (Pennsylvania: IGI Global), 100–113. 10.4018/978-1-5225-9996-8.ch006

[B3] Aguilera R. V. Rupp D. E. Williams C. A. Ganapathi J. (2007). Putting the S back in corporate social responsibility: a multilevel theory of social change in organizations. *Acad. Manage. Rev.* 32 836–863. 10.5465/amr.2007.25275678

[B4] Aguinis H. Glavas A. (2012). What we know and don’t know about corporate social responsibility: a review and research agenda. *J. Manag.* 38 932–968. 10.1177/0149206311436079

[B5] Alarcon G. M. Edwards J. M. (2011). The relationship of engagement, job satisfaction and turnover intentions. *Stress Health* 27 e294–e298. 10.1002/smi.1365

[B6] Ambrose M. L. Seabright M. A. Schminke M. (2002). Sabotage in the workplace: the role of organizational justice. *Organ. Behav. Hum. Decis. Process.* 89 947–965. 10.1016/S0749-5978(02)00037-7

[B7] Antonaki X.-E. Trivellas P. (2014). Psychological contract breach and organizational commitment in the Greek banking sector: the mediation effect of Job satisfaction. *Proc. Soc. Behav. Sci.* 148 354–361. 10.1016/j.sbspro.2014.07.053

[B8] Arnold J. A. Arad S. Rhoades J. A. Drasgow F. (2000). The empowering leadership questionnaire: the construction and validation of a new scale for measuring leader behaviors. *J. Organ. Behav.* 21 249–269. 10.1002/(SICI)1099-1379(200005)21:3<249::AID-JOB10>3.0.CO;2-#

[B9] Ashforth B. E. Mael F. (1989). Social identity theory and the organization. *Acad. Manage. Rev.* 14 20–39. 10.5465/amr.1989.4278999

[B10] Avery D. R. McKay P. F. Wilson D. C. Volpone S. D. Killham E. A. (2011). Does voice go flat? how tenure diminishes the impact of voice. *Hum. Resour. Manag.* 50 147–158. 10.1002/hrm.20403

[B11] Aydogdu S. Asikgil B. (2011). An empirical study of the relationship among job satisfaction, organizational commitment and turnover intention. *Int. Rev. Manage. Market.* 1 43–53.

[B12] Azim M. (2016). Corporate Social Responsibility and employee behavior: mediating role of organizational commitment. *Rev. Bus. Manag.* 18 207–225. 10.7819/rbgn.v18i60.2319

[B13] Azim M. T. Diyab A. A. Al-Sabaan S. A. (2014). CSR, employee job attitude and behavior: Saudi bank experience. *Transylvanian Rev. Adm. Sci.* 43 25–47.

[B14] Babalola M. T. Ren S. Ogbonnaya C. Riisla K. Soetan G. T. Gok K. (2020). Thriving at work but insomniac at home: understanding the relationship between supervisor bottom-line mentality and employee functioning. *Hum. Relat.* 75 33–57. 10.1177/0018726720978687

[B15] Barton H. Barton L. C. (2011). Trust and psychological empowerment in the Russian work context. *Hum. Resour. Manag. Rev.* 21 201–208. 10.1016/j.hrmr.2011.02.001

[B16] Basil D. Z. Erlandson J. (2008). Corporate Social Responsibility website representations: a longitudinal study of internal and external self-presentations. *J. Mark. Commun.* 14 125–137. 10.1080/13527260701858497

[B17] Bedarkar M. Pandita D. (2014). A study on the drivers of employee engagement impacting employee performance. *Proc. Soc. Behav. Sci.* 133 106–115. 10.1016/j.sbspro.2014.04.174

[B18] Bennett R. J. Robinson S. L. (2000). Development of a measure of workplace deviance. *J. Appl. Psychol.* 85 349–60. 10.1037/0021-9010.85.3.349 10900810

[B19] Berry B. (2004). Organizational culture: a framework and strategies for facilitating employee whistleblowing. *Employee Responsib. Rights J.* 16 1–11. 10.1023/B:ERRJ.0000017516.40437.b1

[B20] Blau P. M. (1964). *Exchange And Power In Social Life.* New York: John Wiley & Sons.

[B21] Bright L. (2008). Does public service motivation really make a difference on the job satisfaction and turnover intentions of public employees? *Am. Rev. Public Adm.* 38 149–166. 10.1177/0275074008317248

[B22] Campbell J. L. (2007). Why would Corporations behave in socially responsible ways? An Institutional Theory of Corporate Social Responsibility. *Acad. Manag. Rev.* 32 946–967. 10.5465/amr.2007.25275684

[B23] Chaudhary R. (2017). CSR and turnover intentions: examining the underlying psychological mechanisms. *Soc. Responsib. J.* 13 643–660. 10.1108/SRJ-10-2016-0184

[B24] Chen D. Gao H. Ma Y. (2021). Human capital-driven acquisition: evidence from the inevitable disclosure doctrine. *Manag. Sci.* 67 4643–4664. 10.1287/mnsc.2020.3707 19642375

[B25] Cohen J. (1988). *Statistical Power Analysis for the Behavioral Sciences.* Mahwah: Lawrence Erlbaum.

[B26] Colbert A. E. Mount M. K. Harter J. K. Witt L. A. Barrick M. R. (2004). Interactive effects of personality and perceptions of the work situation on workplace deviance. *J. Appl. Psychol.* 89 599–609. 10.1037/0021-9010.89.4.599 15327347

[B27] Cramer J. (2005). Company Learning about Corporate Social Responsibility. *Bus. Strat. Environ.* 14 255–266. 10.1002/bse.432

[B28] Cranny C. J. Smith P. C. Stone E. (1992). *Job Satisfaction: How People Feel About Their Jobs And How It Affects Their Performance.* Minneapolis: Lexington Books.

[B29] Crant J. Kim T. Wang J. (2011). Dispositional antecedents of demonstration and usefulness of voice behavior. *J. Bus. Psychol.* 26 285–297. 10.1007/s10869-010-9197-y

[B30] Crede M. Chernyshenko O. S. Stark S. Dalal R. S. Bashshur M. (2007). Job satisfaction as mediator: an assessment of job satisfaction’s position within the nomological network. *J. Occup. Organ. Psychol.* 80 515–538. 10.1348/096317906X136180

[B31] Cronbach L. J. (1951). Coefficient alpha and the internal structure of tests. *Psychometrika* 16 297–334. 10.1007/BF02310555

[B32] Cropanzano R. Mitchell M. S. (2005). Social exchange theory: an interdisciplinary review. *J. Manag.* 31 874–900. 10.1177/0149206305279602

[B33] Cropanzano R. Rupp D. E. (2003). An overview of organizational justice: implications for work motivation. *Motiv. Work Behav.* 7 82–95.

[B34] Czarnota-Bojarska J. (2015). Counterproductive work behavior and job satisfaction: a surprisingly rocky relationship. *J. Manag. Organ.* 21 460–470. 10.1017/jmo.2015.15

[B35] Dalal R. S. (2005). A meta-analysis of the relationship between organizational citizenship behavior and counterproductive work behavior. *J. Appl. Psychol.* 90 1241–55. 10.1037/0021-9010.90.6.1241 16316277

[B36] Davis-Blake A. Broschak J. P. George E. (2003). Happy together? how using nonstandard workers affects exit, voice, and loyalty among standard employees. *Acad. Manag. J.* 46 475–485. 10.5465/30040639 30040639

[B37] de Gilder D. Schuyt T. N. M. Breedijk M. (2005). Effects of an employee volunteering program on the work force: the ABN-AMRO Case. *J. Bus. Ethics* 61 143–152. 10.1007/s10551-005-7101-x

[B38] De Roeck K. Delobbe N. (2012). Do environmental CSR initiatives serve organizations’ legitimacy in the oil industry? Exploring employees’ reactions through organizational identification theory. *J. Bus. Ethics* 110 397–412. 10.1007/s10551-012-1489-x

[B39] De Roeck K. Marique G. Stinglhamber F. Swaen V. (2014). Understanding employees’ responses to corporate social responsibility: mediating roles of overall justice and organisational identification. *Int. J. Hum. Resour. Manag.* 25 91–112. 10.1080/09585192.2013.781528

[B40] Deery S. J. Iverson R. D. Buttigieg D. M. Zatzick C. D. (2014). Can union voice make a difference? the effect of union citizenship behavior on employee absence. *Hum. Resour. Manag.* 53 211–228. 10.1002/hrm.21549

[B41] Detert J. R. Burris E. R. (2007). Leadership behavior and employee voice: is the door really open? *Acad. Manag. J.* 50 869–884. 10.5465/amj.2007.26279183

[B42] Dhanesh G. S. (2014). CSR as organization–employee relationship management strategy: a case study of socially responsible information technology companies in India. *Manag. Commun. Q.* 28 130–149. 10.1177/0893318913517238

[B43] Djaelani A. K. Sanusi A. Triatmanto B. (2021). Spiritual leadership, job Satisfaction, and its effect on organizational commitment and organizational citizenship behavior. *Manag. Sci. Lett.* 11 3907–3914. 10.5267/j.msl.2020.7.020

[B44] Edmans A. (2012). The Link Between Job Satisfaction and Firm Value, With Implications for Corporate Social Responsibility. *Acad. Manag. Persp.* 26 1–19. 10.5465/amp.2012.0046

[B45] Ellen P. S. Webb D. J. Mohr L. A. (2006). Building corporate associations: consumer attributions for corporate socially responsible programs. *J. Acad. Mark. Sci.* 34 147–157. 10.1177/0092070305284976

[B46] Engemann K. N. Scott C. W. (2020). Voice in safety-oriented organizations: examining the intersection of hierarchical and mindful social contexts. *Hum. Resour. Manag. Rev.* 30:100650. 10.1016/j.hrmr.2018.05.002

[B47] Farh J. Zhong C. Organ D. W. (2004). Organizational citizenship behavior in the People’s republic of China. *Organ. Sci.* 15 241–253. 10.1287/orsc.1030.0051 19642375

[B48] Farndale E. Van Ruiten J. Kelliher C. Hope-Hailey V. (2011). The influence of perceived employee voice on organizational commitment: an exchange perspective. *Hum. Resour. Manag.* 50 113–129. 10.1002/hrm.20404

[B49] Farooq M. Farooq O. Cheffi W. (2019). How do employees respond to the CSR initiatives of their organizations: empirical evidence from developing countries. *Sustainability* 11:2646. 10.3390/su11092646

[B50] Farooq M. Farooq O. Jasimuddin S. M. (2014). Employees response to corporate social responsibility: exploring the role of employees’ collectivist orientation. *Eur. Manag. J.* 32 916–927. 10.1016/j.emj.2014.03.002

[B51] Farooq O. Payaud M. Merunka D. Valette-Florence P. (2013). The impact of corporate social responsibility on organizational commitment: exploring multiple mediation mechanisms. *J. Bus. Ethics* 125 563–580. 10.1007/s10551-013-1928-3

[B52] Farooq O. Rupp D. E. Farooq M. (2017). The multiple pathways through which internal and external corporate social responsibility influence organizational identification and multifoci outcomes: the moderating role of cultural and social orientations. *Acad. Manag. J.* 60 954–985. 10.5465/amj.2014.0849

[B53] Faul F. Erdfelder E. Lang A. G. Buchner A. (2007). G*Power 3: a flexible statistical power analysis program for the social, behavioral, and biomedical sciences. *Behav. Res. Methods* 39 175–191. 10.3758/BF03193146 17695343

[B54] Folger R. Cropanzano R. (2001). “Fairness theory: justice as accountability,” in *Advances in Organizational Justice*, eds Greenberg J. Cropanzano R. (Stanford: Stanford University Press), 1–55.

[B55] Fornell C. Larcker D. F. (1981). Structural equation models with unobservable variables and measurement error: algebra and statistics. *J. Mark. Res.* 18 382–388. 10.1177/002224378101800313

[B56] Fuller J. B. Barnett T. Hester K. Relyea C. Frey L. (2007). An exploratory examination of voice behavior from an impression management perspective. *J. Manag. Issues* 19 134–151.

[B57] Gao L. Janssen O. Shi K. (2011). Leader trust and employee voice: the moderating role of empowering leader behaviors. *Leadersh. Q.* 22 787–798. 10.1016/j.leaqua.2011.05.015

[B58] Gharleghi B. Afshar Jahanshahi A. Nawaser K. (2018). The outcomes of corporate social responsibility to employees: empirical evidence from a developing country. *Sustainability* 10:698. 10.3390/su10030698

[B59] Gong Y. Chang S. Cheung S. (2010). High-performance work system and collective OCB: a collective social exchange. *Hum. Resour. Manag. J.* 20 119–137. 10.1111/j.1748-8583.2010.00123.x

[B60] Graafland J. Schouten C. M. D. (2012). Motives for corporate social responsibility. *Economist* 160 377–396. 10.1007/s10645-012-9198-5

[B61] Grant A. M. (2013). Rocking the boat but keeping it steady: the role of emotional regulation in employee voice. *Acad. Manag. J.* 56 1703–1723. 10.5465/amj.2011.0035

[B62] Greenidge D. Devonish D. Alleyne P. (2014). The relationship between ability-based emotional intelligence and contextual performance and counterproductive work behaviors: a test of the mediating effects of job satisfaction. *Hum. Perform.* 27 225–242. 10.1080/08959285.2014.913591

[B63] Griep Y. Vantilborgh T. Jones S. K. (2020). The relationship between psychological contract breach and counterproductive work behavior in social enterprises: do paid employees and volunteers differ? *Econ. Industr. Democr.* 41 727–745. 10.1177/0143831X17744029

[B64] Guo X. W. (2012). Counterproductive work behaviors, Confucian values, and production deviance: the mediating effect of job satisfaction. *Soc. Behav. Pers. Int. J.* 40 1045–1056. 10.2224/sbp.2012.40.6.1045

[B65] Hackman J. R. Oldham G. R. (1975). Development of the job diagnostic survey. *J. Appl. Psychol.* 60 159–170. 10.1037/h0076546

[B66] Hager W. (2006). Die Fallibilität empirischer Daten und die Notwendigkeit der Kontrolle von falschen Entscheidungen [The fallibility of empirical data and the need for controlling for false decisions]. *Z. Psychol.* 214 10–23. 10.1026/0044-3409.214.1.10

[B67] Hamid N. (2020). The application of spiritual leadership affects job satisfaction and deviant behavior in the workplace. *Hum. Syst. Manag.* 39 1–11. 10.3233/HSM-190639

[B68] Hansen S. D. Dunford B. B. Boss A. D. Boss R. W. Angermeier I. (2011). Corporate social responsibility and the benefits of employee trust: a cross-disciplinary perspective. *J. Bus. Ethics* 102 29–45. 10.1007/s10551-011-0903-0

[B69] Hardy G. E. Woods D. Wall T. D. (2003). The impact of psychological distress on absence from work. *J. Appl. Psychol.* 88 306–314. 10.1037/0021-9010.88.2.306 12731714

[B70] He P. Jiang C. Xu Z. Shen C. (2021). Knowledge hiding: current research status and future research directions. *Front. Psychol.* 12:748237. 10.3389/fpsyg.2021.748237 34777143 PMC8586422

[B71] Hogg M. A. Terry D. I. (2000). Social identity and self-categorization processes in organizational contexts. *Acad. Manag. Rev.* 25 121–140. 10.5465/amr.2000.2791606

[B72] Hsiung H. (2012). Authentic leadership and employee voice behavior: a multi-level psychological process. *J. Bus. Ethics* 107 349–361. 10.1007/s10551-011-1043-2

[B73] Huang M. H. Cheng Z. H. (2012). The effects of inter-role conflicts on turnover intentions among frontline service providers: does gender matter? *Serv. Industr. J.* 32 367–381. 10.1080/02642069.2010.545391

[B74] Illies R. Wilson K. S. Wagner D. T. (2009). The spillover of daily job satisfaction onto employees’ family lives: the facilitating role of work-family integration. *Acad. Manag. J.* 52 87–102. 10.5465/amj.2009.36461938

[B75] Janssen O. de Vries T. Cozijnsen A. J. (1998). Voicing by adaptive and innovating employees: an empirical study on how personality and environment interact to affect voice behavior. *Hum. Relat.* 51 945–967. 10.1177/001872679805100705

[B76] Jones D. A. (2010). Does serving the community also serve the company? Using organizational identification and social exchange theories to understand employee responses to a volunteerism programme. *J. Occup. Organ. Psychol.* 83 857–878. 10.1348/096317909X477495

[B77] Joo B. Park S. (2010). Career satisfaction, organizational commitment, and turnover intention: the effects of goal orientation, organizational learning culture and developmental feedback. *Leadersh. Organ. Dev. J.* 31 482–500. 10.1108/01437731011069999

[B78] Judge T. A. Bono J. E. Locke E. A. (2000). Personality and job satisfaction: the mediating role of job characteristics. *J. Appl. Psychol.* 85 237–249. 10.1037/0021-9010.85.2.237 10783540

[B79] Judge T. A. Scott B. A. Ilies R. (2006). Hostility, job attitudes, and workplace deviance: test of a multilevel model. *J. Appl. Psychol.* 91 126–38. 10.1037/0021-9010.91.1.126 16435943

[B80] Khatri N. Fern C. T. Budhwar P. (2001). Explaining employee turnover in an Asian context. *Hum. Resour. Manag. J.* 11 54–74. 10.1111/j.1748-8583.2001.tb00032.x

[B81] Kim H. L. Rhou Y. Uysal M. Kwon N. (2017). An examination of the links between corporate social responsibility (CSR) and its internal consequences. *Int. J. Hosp. Manag.* 61 26–34. 10.1016/j.ijhm.2016.10.011

[B82] Kim J. S. Song H. J. Lee C. K. (2016). Effects of corporate social responsibility and internal marketing on organizational commitment and turnover intentions. *Int. J. Hosp. Manag.* 55 25–32. 10.1016/j.ijhm.2016.02.007

[B83] Kline R. (2011). *Principles and Practice of Structural Equation Modeling.* New York: Guilford Press.

[B84] Ko S. H. Moon T. W. Hur W. M. (2018). Bridging service employees’ perceptions of CSR and organizational citizenship behavior: the moderated mediation effects of personal traits. *Curr. Psychol.* 37 816–831. 10.1007/s12144-017-9565-0

[B85] Lacobucci D. Saldanha N. Deng X. (2007). A meditation on mediation: evidence that structural equations models perform better than regressions. *J. Consum. Psychol.* 17 139–153. 10.1016/S1057-7408(07)70020-7

[B86] Landau J. (2009). To speak or not to speak: predictors of voice propensity. *J. Organ. Cult. Commun. Confl.* 13 35–54.

[B87] LePine J. A. Van Dyne L. (1998). Predicting voice behavior in work groups. *J. Appl. Psychol.* 83 853–868. 10.1037/0021-9010.83.6.853

[B88] Li N. Zhang L. Xiao G. Chen J. Lu Q. (2019). The relationship between workplace violence, job satisfaction, and turnover intention in emergency nurses. *Int. Emerg. Nurs.* 45 50–55. 10.1016/j.ienj.2019.02.001 30797732

[B89] Liang J. Farh C. I. Farh J.-L. (2012). Psychological antecedents of promotive and prohibitive voice: a two-wave examination. *Acad. Manag. J.* 55 71–92. 10.5465/amj.2010.0176

[B90] Liu W. Zhu R. Yang Y. (2010). I warn you because I like you: voice behavior, employee identifications, and transformational leadership. *Leadersh. Q.* 21 189–202. 10.1016/j.leaqua.2009.10.014

[B91] Lu A. C. C. Gursoy D. (2013). Impact of job burnout on satisfaction and turnover intention: do generational differences matter? *J. Hosp. Tour. Res.* 40 210–235. 10.1177/1096348013495696

[B92] Lu L. Lu A. C. C. Gursoy D. Neale N. R. (2016). Work engagement, job satisfaction, and turnover intentions: a comparison between supervisors and line-level employees. *Int. J. Contemp. Hosp. Manag.* 28 737–761. 10.1108/IJCHM-07-2014-0360

[B93] Mael F. Ashforth B. E. (1992). Alumni and their alma mater: a partial test of the reformulated model of organizational identification. *J. Organ. Behav.* 13 103–123. 10.1002/job.4030130202

[B94] Mahmood F. Qadeer F. Abbas Z. Hussain I. Saleem M. Hussain A. (2020). Corporate social responsibility and employees’ negative behaviors under abusive supervision: a multilevel insight. *Sustainability* 12:2647. 10.3390/su12072647

[B95] Maignan I. Ferrell O. C. (2000). Measuring corporate citizenship in two countries: the case of the United States and France. *J. Bus. Ethics* 23 283–297. 10.1023/A:1006262325211

[B96] Malhotra M. Kathuria K. (2017). Relationship between spiritual intelligence, job satisfaction and counterproductive work behaviour among employees of multinational companies in India. *J. Psychosoc. Res.* 12 315–323.

[B97] Mallory D. B. Rupp D. E. (2016). “”Good” leadership: using corporate social responsibility to enhance leader-member exchange,” in *The Oxford Handbook Of Leader-Member Exchange*, eds Bauer T. N. Erdogan B. (Oxford: Oxford University Press), 335–350.

[B98] Manika D. Gregory-Smith D. Wells V. K. Comerford L. Aldrich-Smith L. (2017). Linking environmental sustainability and healthcare: the effects of an energy-saving intervention in two hospitals. *Int. J. Bus. Sci. Appl. Manag.* 11 32–55.

[B99] Marquis C. Qian C. (2014). Corporate social responsibility reporting in China: symbol or substance? *Organ. Sci.* 25 127–148. 10.1287/orsc.2013.0837 19642375

[B100] Mashi M. S. (2018). The mediating role of job satisfaction in the relationship between organizational justice and employee outcomes. *Int. J. Public Adm.* 41 1351–1360. 10.1080/01900692.2017.1388819

[B101] Matten D. Moon J. (2008). Implicit” and “Explicit” CSR: a conceptual framework for a comparative understanding of Corporate Social Responsibility. *Acad. Manag. Rev.* 33 404–424. 10.5465/amr.2008.31193458

[B102] Maynes T. D. Podsakoff P. M. (2014). Speaking more broadly: an examination of the nature, antecedents, and consequences of an expanded set of employee voice behaviors. *J. Appl. Psychol.* 99 87–112. 10.1037/a0034284 24041119

[B103] McNall L. A. Masuda A. D. Nicklin J. M. (2009). Flexible work arrangements, job satisfaction, and turnover intentions: the mediating role of work-to-family enrichment. *J. Psychol.* 144 61–81. 10.1080/00223980903356073 20092070

[B104] McWilliams A. Siegel D. (2001). Corporate social responsibility: a theory of the firm perspective. *Acad. Manag. Rev.* 26 117–127. 10.5465/amr.2001.4011987

[B105] Meier L. L. Spector P. E. (2013). Reciprocal effects of work stressors and counterproductive work behavior: a five-wave longitudinal study. *J. Appl. Psychol.* 98 529–539. 10.1037/a0031732 23379915

[B106] Memon K. R. (2014b). Strategic role of HRD in employee skill development: an employer perspective. *J. Hum. Resour. Manag.* 2 27–32. 10.11648/j.jhrm.20140201.15

[B107] Memon K. R. (2014a). Effects of leadership styles on employee performance: integrating the mediating role of culture, gender and moderating role of communication. *Int. J. Manag. Sci. Bus. Res.* 3 63–80.

[B108] Memon K. R. Ghani B. (2020). The relationship between psychological contract and voice behavior—a social exchange perspective. *Asian J. Bus. Ethics* 9 257–274. 10.1007/s13520-020-00109-4

[B109] Memon K. R. Ghani B. Kazi A. A. (2018). Restructuring the relationship between performance management and employee engagement. *Pakistan Bus. Rev.* 20 99–109.

[B110] Memon K. R. Ghani B. Khalid S. (2020). The relationship between corporate social responsibility and employee engagement - A social exchange perspective. *Int. J. Bus. Sci. Appl. Manag.* 15 1–16.

[B111] Memon K. R. Say K. O. Khalid S. Ghani B. (2021). Mediation - Moderation mechanism between the relationship of corporate social responsibility and employees’ promotive voice behavior. *Int. J. Bus. Sci. Appl. Manag.* 16 50–68.

[B112] Moro S. Ramos R. F. Rita P. (2020). What drives job satisfaction in IT companies? *Int. J. Product. Perform. Manag.* 70 391–407. 10.1108/IJPPM-03-2019-0124

[B113] Morrison E. W. (2011). Employee voice behavior: integration and directions for future research. *Acad. Manag. Ann.* 5 373–412. 10.5465/19416520.2011.574506

[B114] Mount M. Ilies R. Johnson E. (2006). Relationship of personality traits and counterproductive work behaviors: the mediating effects of job satisfaction. *Pers. Psychol.* 59 599–622. 10.1111/j.1744-6570.2006.00048.x

[B115] Ng T. W. Yam K. C. Aguinis H. (2019). Employee perceptions of corporate social responsibility: effects on pride, embeddedness, and turnover. *Pers. Psychol.* 72 107–137. 10.1111/peps.12294

[B116] Ng T. W. H. Feldman D. C. (2011). Employee voice behavior: a meta-analytic test of the conservation of resources framework. *J. Organ. Behav.* 33 216–234. 10.1002/job.754

[B117] Nikolaou I. Vakola M. Bourantas D. (2008). Who speaks up at work? Dispositional influences on employees’ voice behavior. *Pers. Rev.* 37 666–679. 10.1108/00483480810906892

[B118] Nunnally J. C. (1978). *Psychometric Theory*, 2nd Edn. New York: McGraw-Hill.

[B119] Ooi S. K. Ooi C. A. Memon K. R. (2020). The role of CSR oriented organisational culture in eco-innovation practices. *World Rev. Entrepreneursh. Manag. Sustain. Dev.* 16 538–556. 10.1504/WREMSD.2020.110451

[B120] Organ D. W. Ryan K. (1995). A meta-analytic review of attitudinal and dispositional predictors of organizational citizenship behavior. *Pers. Psychol.* 48 775–802. 10.1111/j.1744-6570.1995.tb01781.x

[B121] Oshagbemi T. (2013). *Job Satisfaction In Higher Education.* Bloomington: Trafford Publishing.

[B122] Pham H. (2020). Impact of human resource management practices on enterprises’ competitive advantages and business performance: evidence from telecommunication industry. *Manag. Sci. Lett.* 10 721–732. 10.5267/j.msl.2019.10.025

[B123] Qin X. Direnzo M. S. Xu M. Duan Y. (2014). When do emotionally exhausted employees speak up? exploring the potential curvilinear relationship between emotional exhaustion and voice. *J. Organ. Behav.* 35 1018–1041. 10.1002/job.1948

[B124] Randhawa G. (2007). Relationship between job satisfaction and turnover intentions: an empirical analysis. *Indian Manag. Stud. J.* 11 149–159.

[B125] Rees C. Alfes K. Gatenby M. (2013). Employee voice and engagement: connections and consequences. *Int. J. Hum. Resour. Manag.* 24 2780–2798. 10.1080/09585192.2013.763843

[B126] Riordan C. M. Gatewood R. D. Bill J. B. (1997). Corporate image: employee reactions and implications for managing corporate social performance. *J. Bus. Ethics* 16 401–412. 10.1023/A:1017989205184

[B127] Rousseau D. M. (1989). Psychological and implied contracts in organizations. *Employee Responsib. Rights J.* 2 121–139. 10.1007/BF01384942

[B128] Ruiz-Palomino P. Martínez-Cañas R. (2014). Ethical culture, ethical intent, and organizational citizenship behavior: the moderating and mediating role of person-organization fit. *J. Bus. Ethics* 120 95–108. 10.1007/s10551-013-1650-1

[B129] Rupp D. E. Ganapathi J. Aguilera R. V. Williams C. A. (2006). Employee reactions to corporate social responsibility: an organizational justice framework. *J. Organ. Behav.* 27 537–543. 10.1002/job.380

[B130] Rupp D. E. Mallory D. B. (2015). Corporate social responsibility: psychological, person-centric, and progressing. *Annu. Rev. Organ. Psychol. Organ. Behav.* 2 211–236. 10.1146/annurev-orgpsych-032414-111505

[B131] Rupp D. E. Skarlicki D. Shao R. (2013). The psychology of corporate social responsibility and humanitarian work: a person-centric perspective. *Industr. Organ. Psychol.* 6 361–368. 10.1111/iops.12068

[B132] Sackett P. R. (2002). The structure of counterproductive work behaviors: dimensionality and relationships with facets of job performance. *Int. J. Select. Assess.* 10 5–11. 10.1111/1468-2389.00189

[B133] Seibert S. E. Wang G. Courtright S. H. (2011). Antecedents and consequences of psychological and team empowerment in organizations: a meta-analytic review. *J. Appl. Psychol.* 96 981–1003. 10.1037/a0022676 21443317

[B134] Shah I. H. Ajmal M. Rahman F. (2010). Structure of technical education and vocational training in Pakistan. *J. Tech. Educ. Train.* 2 63–76.

[B135] Sherf E. N. Parke M. R. Isaakyan S. (2020). Distinguishing voice and silence at work: unique relationships with perceived impact, psychological safety, and burnout. *Acad. Manag. J.* 64 114–148. 10.5465/amj.2018.1428

[B136] Shin I. Hur W. M. Kim M. Kang S. (2017). Hidden roles of CSR: perceived corporate social responsibility as a preventive against counterproductive work behaviors. *Sustainability* 9:955. 10.3390/su9060955

[B137] Shiun T. Ho A. (2012). *The Impact of Perceived CSR on Employee Performance and Turnover Intention: An Examination of the Mediating Effect of Organizational Justice and Organization-Based Self esteem.* Ph.D. thesis. Singapore: Singapore Management University.

[B138] Si S. Li Y. (2012). Human resource management practices on exit, voice, loyalty, and neglect: organizational commitment as a mediator. *Int. J. Hum. Resour. Manag.* 23 1705–1716. 10.1080/09585192.2011.580099

[B139] Singh S. K. Singh A. P. (2019). Interplay of organizational justice, psychological empowerment, organizational citizenship behavior, and job satisfaction in the context of circular economy. *Manag. Decis.* 57 937–952. 10.1108/MD-09-2018-0966

[B140] Singhapakdi A. Sirgy M. J. Lee D. J. Senasu K. Yu G. B. Nisius A. M. (2014). Gender disparity in job satisfaction of Western versus Asian managers. *J. Bus. Res.* 67 1257–1266. 10.1016/j.jbusres.2013.04.004

[B141] Skudiene V. Auruskeviciene V. (2012). The contribution of corporate social responsibility to internal employee motivation. *Baltic J. Manag.* 7 49–67. 10.1108/17465261211197421

[B142] Srivastava S. Agrawal S. (2020). Resistance to change and turnover intention: a moderated mediation model of burnout and perceived organizational support. *J. Organ. Change Manag.* 33 1431–1447. 10.1108/JOCM-02-2020-0063

[B143] Stamper C. L. Van Dyne L. (2001). Work status and organizational citizenship behavior: a field study of restaurant employees. *J. Organ. Behav.* 22 517–536. 10.1002/job.100

[B144] Tajfel H. Turner J. C. (1979). “An Integrative Theory of Intergroup Conflict,” in *The Social Psychology of Intergroup Relations*, eds Austin W. G. Worchel S. (Monterey: Brooks/Cole Publishing Co), 33–47.

[B145] Tajfel H. Turner J. C. (1986). “The social identity theory of intergroup behavior,” in *Psychology Of Intergroup Relations*, eds Worchel S. Austin W. (Chicago: Nelson Hall), 7–24.

[B146] Tangirala S. Ramanujam R. (2008). Exploring nonlinearity in employee voice: the effects of personal control and organizational identification. *Acad. Manag. J.* 51 1189–1203. 10.5465/amj.2008.35732719

[B147] Turker D. (2009). How corporate social responsibility influences organizational commitment. *J. Bus. Ethics* 89 189–204. 10.1007/s10551-008-9993-8

[B148] Turnley W. H. Bolino M. C. Lester S. W. Bloodgood J. M. (2003). The Impact of psychological contract fulfillment on the performance of in-role and organizational citizenship behaviors. *J. Manag.* 29 187–206. 10.1177/014920630302900204

[B149] Tziner A. Oren L. Bar Y. Kadosh G. (2011). Corporate social responsibility, organizational justice and job satisfaction: how do they interrelate, if at all? *J. Work Organ. Psychol.* 27 67–72. 10.5093/tr2011v27n1a7

[B150] Ugwu F. O. Onyishi I. E. Rodríguez-Sánchez A. M. (2014). Linking organizational trust with employee engagement: the role of psychological empowerment. *Pers. Rev.* 43 377–400. 10.1108/PR-11-2012-0198

[B151] Valentine S. Fleischman G. (2008). Ethics programs, perceived corporate social responsibility and job satisfaction. *J. Bus. Ethics* 77 159–172. 10.1007/s10551-006-9306-z

[B152] Viseu J. Pinto P. Borralha S. de Jesus S. N. (2020). Role of individual and organizational variables as predictors of job satisfaction among hotel employees. *Tour. Hosp. Res.* 20:146735842092406. 10.1177/1467358420924065

[B153] Walumbwa F. O. Schaubroeck J. (2009). Leader personality traits and employee voice behavior: mediating roles of ethical leadership andwork group psychological safety. *J. Appl. Psychol.* 94 1275–1286. 10.1037/a0015848 19702370

[B154] Wang W. Fu Y. Qiu H. Moore J. H. Wang Z. (2017). Corporate social responsibility and employee outcomes: a moderated mediation model of organizational identification and moral identity. *Front. Psychol.* 8:1906. 10.3389/fpsyg.2017.01906 29163287 PMC5671997

[B155] Wenbin W. Fengjun L. Hui L. (2012). Effect of corporate social responsibility on organizational citizenship behavior from the perspective of the reciprocity theory. *Contemp. Econ. Manag.* 11:006.

[B156] Wu W. Tang F. Dong X. Liu C. (2015). Different identifications cause different types of voice: a role identity approach to the relations between organizational socialization and voice. *Asia Pacif. J. Manag.* 32 251–287. 10.1007/s10490-014-9384-x

[B157] Yu Y. Choi Y. (2014). Corporate social responsibility and firm performance through the mediating effect of organizational trust in Chinese firms. *Chin. Manag. Stud.* 8 577–592. 10.1108/CMS-10-2013-0196

[B158] Zhang L. Deng Y. (2016). Guanxi with supervisor and counterproductive work behavior: the mediating role of job satisfaction. *J. Bus. Ethics* 134 413–427. 10.1007/s10551-014-2438-7

[B159] Zhang X. Hu B. Qiu M. (2014). Job satisfaction as a mediator in the relationship between performance appraisal and voice behavior. *Soc. Behav. Pers.* 42 1315–1324. 10.2224/sbp.2014.42.8.1315

[B160] Zhao H. Zhou Q. He P. Jiang C. (2019). How and when does socially responsible HRM affect employees’ organizational citizenship behaviors toward the environment? *J. Bus. Ethics* 169 371–385. 10.1007/s10551-019-04285-7

[B161] Zheng Q. Luo Y. Maksimov V. (2014). Achieving legitimacy through corporate social responsibility: the case of emerging economy firms. *J. World Bus.* 50 389–403. 10.1016/j.jwb.2014.05.001

[B162] Zhuang M. Zhu W. Huang L. Pan W.-T. (2021). Research of influence mechanism of corporate social responsibility for smart cities on consumers’ purchasing intention. *Library Hi Tech* [Epub Online ahead of Print]. 10.1108/LHT-11-2020-0290

[B163] Zulfiqar S. Sadaf R. Popp J. Vveinhardt J. Máté D. (2019). An examination of corporate social responsibility and employee behavior: the case of Pakistan. *Sustainability* 11:3515. 10.3390/su11133515

