# Supplementary material for: Linking Corporate Social Responsibility to Workplace Deviant Behaviors: Mediating Role of Job Satisfaction
Source: Front Psychol. 2021 Dec 30;12:803481. doi: 10.3389/fpsyg.2021.803481 (PMC8755641; doi:10.3389/fpsyg.2021.803481)
Supplement: Supplementary file 1 [file Data_Sheet_1.docx]

**Appendix 1**

**Measurement instrument items, loadings, Cronbach’s @**

| **Construct/Latent Variable** | **Items** | **Loadings** | **@** | **Source** |
| --- | --- | --- | --- | --- |
| **External**  **CSR** | My company participates to the activities which aim to protect and improve the quality of the natural.  My company makes investment to create a better life for the future generations  My company implements special programs to minimize its negative impact on the natural environment  My company targets a sustainable growth which considers to the future generations  My company gives adequate contributions to charities.  My company supports the non-governmental organizations working in the problematic areas  My company contributes to the campaigns and projects that promote the well-being of the society  My company protects consumer rights beyond the legal requirements  My company provides full and accurate information about its products to its customers  Customer satisfaction is highly important for my company | 0.845  0.857  0.467  0.486  0.884  0.499  0.812  0.498  0.794  0.842 | **0.79** | M.Farooq et al (2014)  O.Farooq et al (2013) |
| **Internal**  **CSR** | My company encourages its employees to participate to the voluntary activities  My company policies encourage the employees to develop their skills and careers  The management of my company primarily concerns with employees’ needs and wants  My company implements flexible policies to provide a good work and life balance for its employees  The managerial decisions related with the employees are usually fair  My company supports employees who want to acquire additional education | 0.499  0.854  0.724  0.708  0.821  0.745 | **0.90** | M.Farooq et al (2014)  O.Farooq et al (2013) |
| **Job Satisfaction** | Overall, I am very satisfied with this job.  Overall, I am satisfied with the kind of work I do in this job.  I frequently think of quitting this job.  Most people on this job are very satisfied with the job.  People on this job often think of quitting | 0.789  0.704  0.786  0.499  0.701 | **0.86** | Zhang et al, 2014 |
| **Defensive voice** | I stubbornly argue against changing work methods, even when the proposed changes have merit.  I speak out against changing work policies, even when making changes would be for the best.  I vocally oppose changing how things are done, even when changing is inevitable.  I rigidly argue against changing work procedures, even when implementing the changes makes sense.  I vocally argue against changing work practices, even when making the changes is necessary. | 0.892  0.92  0.90  0.886  0.486 | **0.84** | Maynes and Podsakoff (2014) |
| **Destructive voice** | I often bad-mouth the organization’s policies or objectives.  I often make insulting comments about work-related programs or initiatives.  I frequently make overly critical comments regarding how things are done in the organization.  I often make overly critical comments about the organization’s work practices or methods.  I harshly criticize the organization’s policies, even though the criticism is unfounded. | 0.92  0.90  0.94  0.894  0.828 | **0.82** | Maynes and Podsakoff (2014) |
| **Interpersonal Deviance** | Made fun of someone at work  Said something hurtful to someone at work  Made an ethnic, religious, or racial remark at work  Cursed at someone at work  Played a mean prank on someone at work  Acted rudely toward someone at work  Publicly embarrassed someone at work | 0.489  0.704  0.886  0.799  0.721  0.485  0.704 | **0.92** | Bennett and Robinson (2000) |
| **Organizational Deviance** | Taken property from work without permission  Spent too much time fantasizing or daydreaming instead of working  Falsified a receipt to get reimbursed for more money than you spent on business expenses  Taken an additional or longer break than is acceptable at your workplace  Come in late to work without permission  Littered your work environment  Neglected to follow your boss's instructions  Intentionally worked slower than you could have worked  Discussed confidential company information with an unauthorized person  Used an illegal drug or consumed alcohol on the job  Put little effort into your work  Dragged out work in order to get overtime | 0.855  0.866  0.488  0.489  0.484  0.599  0.712  0.498  0.694  0.942  0.704  0.468 | **0.91** | Bennett and Robinson (2000) |
| **Turnover Intention** | I will likely actively look for a new job in the next year,  I often think about quitting, and  I probably look for a new job in the next year. | 0.865  0.745  0.706 | **0.85** | Khatri et al (2001) |
